# Alterations in electrophysiological indices of perceptual processing and discrimination are associated with co-occurring emotional and behavioural problems in adolescents with autism spectrum disorder

**DOI:** 10.1186/s13229-018-0236-2

**Published:** 2018-10-05

**Authors:** Virginia Carter Leno, Susie Chandler, Pippa White, Isabel Yorke, Tony Charman, Andrew Pickles, Emily Simonoff

**Affiliations:** 1 Institute of Psychiatry, Psychology & Neuroscience, King’s College London, 16 De Crespigny Park, London, SE5 8AF UK; 2 South London and Maudsley NHS Foundation Trust (SLaM), London, UK

**Keywords:** IAMHealth, EEG, ERP, ASD, Perceptual processing, Sensory, Comorbidity, Psychopathology

## Abstract

**Background:**

Many young people with autism spectrum disorder (ASD) experience emotional and behavioural problems. However, the causes of these co-occurring difficulties are not well understood. Perceptual processing atypicalities are also often reported in individuals with ASD, but how these relate to co-occurring emotional and behavioural problems remains unclear, and few studies have used objective measurement of perceptual processing.

**Methods:**

Event-related potentials (ERPs) were recorded in response to both standard and deviant stimuli (which varied in pitch) in an auditory oddball paradigm in adolescents (mean age of 13.56 years, SD = 1.12, range = 11.40–15.70) with ASD (*n* = 43) with a wide range of IQ (mean IQ of 84.14, SD = 24.24, range 27–129). Response to deviant as compared to standard stimuli (as indexed by the mismatch negativity (MMN)) and response to repeated presentations of standard stimuli (habituation) were measured. Multivariate regression tested the association between neural indices of perceptual processing and co-occurring emotional and behavioural problems.

**Results:**

Greater sensitivity to changes in pitch in incoming auditory information (discrimination), as indexed by increased MMN amplitude, was associated with higher levels of parent-rated behaviour problems. MMN amplitude also showed a trend positive correlation with parent-rated sensory hyper-sensitivity. Conversely, greater habituation at the later N2 component was associated with higher levels of emotional problems. Upon more detailed analyses, this appeared to be driven by a selectively greater ERP response to the first (but not the second or third) standard stimuli that followed deviant stimuli. A similar pattern of association was found with other measures of anxiety. All results remained in covariation analyses controlling for age, sex and IQ, although the association between MMN amplitude and behaviour problems became non-significant when controlling for ASD severity.

**Conclusions:**

Findings suggest that alterations in mechanisms of perceptual processing and discrimination may be important for understanding co-occurring emotional and behavioural problems in young people with ASD.

**Electronic supplementary material:**

The online version of this article (10.1186/s13229-018-0236-2) contains supplementary material, which is available to authorized users.

## Background

Co-occurring psychopathology is highly prevalent in children and adolescents with ASD [1–6]; however, the aetiology of these additional emotional and behavioural problems in ASD is not well known. Rates of psychopathology are higher in ASD populations as compared to populations of individuals with intellectual disability (ID) [7, 8], suggesting that ASD is a risk factor, over and above having ID. One approach is to test whether performance in certain cognitive domains, thought to be impaired in individuals with ASD, is also associated with the presence of psychopathology. This will inform future longitudinal studies, where the predictive role of domains can be fully tested. Understanding ASD-specific risk factors will allow novel, targeted interventions to be developed, promoting improved quality of life and better long-term outcomes.

At a group level, ASD is characterised by specific neurocognitive impairments, thought to contribute to the core symptoms of social communication difficulties and restricted, repetitive behaviours [9, 10]. However, few studies have considered how variability in these domains of cognitive functioning may also be important in understanding the preponderance of additional psychopathology in individuals with ASD. The current manuscript focuses upon the domain of sensory or perceptual processing, where individuals with ASD often show atypical functioning. Previous research has highlighted that individuals with ASD experience both hypo- and hyper-sensitivity to perceptual inputs from auditory, tactile and visual sources [11–15] and that alterations in underlying neural processes may underpin these atypical perceptual experiences [16–18]. One of the most well-studied neural indices of perceptual processing in electroencephalography (EEG) paradigms is the mismatch negativity (MMN) component [19]. This is a fronto-central negative component found around 100–200 ms after stimulus presentation, which, in typically developing individuals, is of greater amplitude in response to deviant, as compared to standard stimuli. As MMN amplitude is found to be associated with individual discrimination skill [19–21], some have suggested it is an index of individual sensitivity to changes in incoming information (i.e., discrimination).

In terms of MMN alterations in individuals with ASD, findings are mixed (for a review see [22]). Some have found increased MMN amplitude in individuals with ASD [23–25], and decreased latency [26, 27], which have been interpreted as indexing hyper-sensitivity to unpredictable changes [17]. However, others have found decreased MMN amplitude [28–31] and increased MMN latency [32]. Furthermore, some have reported an association between MMN attenuation and higher sensory sensitivity scores [28, 31]. Differences in findings may be due to variation in the samples (e.g., with/without concurrent ID, older vs. younger children) and experimental paradigms used, as one study found attenuated MMN in children with ASD during non-attended conditions, but when participants were instructed to listen to the sounds, there was no difference between the ASD and typically developing group [33].

Another, albeit less researched area of perceptual processing in ASD is that of habituation. In the types of oddball paradigms used to study discrimination between deviant and standard stimuli reviewed above, one can also study habituation to the standard stimuli, where the neural response exponentially decreases over repeated presentations of the same stimuli. This is thought to allow the brain to filter out irrelevant repetitive stimuli and conserve attentional resources [34]. Research has found reduced neural habituation to repeated presentations of the same stimuli in individuals with ASD [35, 36], and in 9-month old infants at higher genetic risk of developing ASD [37], and some suggest that this reduced habituation may underlie both the hypo- and hyper-sensitivity to sensory input found in individuals with ASD [37].

Although no study has directly looked at how neural indices of perceptual processing are related to emotional and behavioural problems in ASD, there are a small number of studies that used questionnaire measures of sensory/perceptual processing. A small sample pilot study (*n* = 22) found that caregiver-rated sensory processing atypicalities were significantly correlated (*r* = 0.49) with behavioural problems in children with ASD [38]. Another study of young children with ASD found parent-rated sensory avoidance was significantly associated with internalising problems, whereas sensory sensitivity was significantly associated with externalising problems [39]. Similar associations were found in a study that used teacher-rated questionnaires, where a significant correlation was found between tactile and movement sensitivity, and oppositional behaviour in children with ASD [40]. However, the specificity of this association was unclear, as tactile sensitivity was also correlated with ADHD-type symptoms. In the same study, the authors also found an association between difficulties with auditory filtering and internalising problems. A number of studies have reported an association between parent-rated sensory hyper-sensitivity and anxiety symptoms in individuals with ASD [41–44], including one that used physiological reactivity to a sensory challenge as an index of sensitivity [41]. One longitudinal study of toddlers with ASD found sensory over-sensitivity predicted increases in anxiety over and above child age, ASD symptom severity, cognitive ability, and maternal anxiety, but anxiety did not predict changes in sensory over-sensitivity [45], suggesting a potential causal pathway between sensory processing atypicalities and anxiety in ASD.

No study has specifically explored the association between habituation and co-occurring emotional and behavioural problems in individuals with ASD. However, in typically developing adolescents, decreased neural habituation was found to be associated with higher levels of trait anxiety [46]. In terms of how habituation could theoretically relate to anxiety, impaired habituation may lead to repeated and predictable perceptual inputs being experienced as novel and unpredictable, and neuroimaging research has found temporally unpredictable stimuli provoke anxiety behaviours in mice and humans [47].

### Aims

In summary, it appears that individuals with ASD are characterised not only by alterations in neural response to deviant stimuli, but also by decreased habituation to repeated presentation of the same stimuli. Questionnaire studies from individuals with ASD and neuroimaging studies from typically developing individuals suggest that both of these domains may be linked to emotional and behavioural problems. However, no study has specifically tested how neural indices of perceptual processing relate to emotional and behavioural problems in individuals with ASD. The aim of this study was to investigate whether neural responses to (a) deviant vs. standard stimuli and (b) repeated presentation of the standard stimuli were associated with co-occurring emotional and behavioural problems in adolescents with ASD. Based on prior literature, it was hypothesised that greater sensitivity to changes in perceptual information, as indexed by increased MMN amplitude, would be associated with higher levels of emotional and behaviour problems. In terms of habituation, it was hypothesised that decreased habituation would be associated with increased emotional difficulties. Finally, correlations between neural measures of perceptual processing and parent-rated sensory sensitivities were calculated to understand how the selected neural measures related to real-life sensory behaviours.

## Methods

### Participants

Forty-three adolescents with ASD, consisting of 29 males and 14 females, with a mean age of 13.56 years (SD = 1.12, range = 11.40–15.70) and mean IQ of 84.14 (SD = 24.24, range 27–129; *n* = 3 with IQ < 50) completed an auditory oddball paradigm. Participants were part of the QUEST follow-up study, a longitudinal community sample recruited at age 4–8 years [3], which in turn was part of the wider IAMHealth project (https://iamhealthkcl.net//). The target population for the study was all children born between September 01, 2000, and August 31, 2004, living in two London boroughs (one inner and one outer London), who had a clinical diagnosis of ASD. More information about the sampling structure is given in Additional file 1. Although participants had a clinical diagnosis of ASD, the ‘intensively studied’ (hereafter intensive) group (*n* = 83) included at present had their diagnosis confirmed at age 10–16 years with the Autism Diagnostic Observation Schedule-2 (ADOS-2) ([48]) and a subset also with the Autism Diagnostic Interview-Revised (ADI-R) [49]. Both the recommended autism cutoff [49] and the recommended ASD cutoff [50] were applied to the ADI-R data. All participants were above threshold on either or both instruments. Participants in the intensive group were selected to over-represent females, as one of the main aims of the study included sex comparisons. This sample completed a selection of neurocognitive assessments and parent-rated questionnaires. The larger ‘extensively studied’ (extensive) sample (*n* = 128) only completed a selection of parent-rated questionnaires online. The extensive sample did not complete any neurocognitive assessments, but for the purpose of this paper were included to allow for examination of the psychometric properties of the Sensory Experiences Questionnaire—brief version (see below for further details). From the original intensive QUEST sample (*n* = 83, which had an IQ range of 19–120), only those who were able to complete the auditory oddball paradigm (*n* = 43) were included in present analyses. All participating families gave their written informed consent, and the study was approved by Camden and King’s Cross Ethics Sub-Committee (14/LO/2098). Table 1 gives demographic information for the sample, and comparison of key outcome measures between the total sample (intensive + extensive combined), the intensive sample and those who completed the auditory oddball paradigm is given in Additional file 1. All participating families gave their written informed consent.

**Table 1:** Sample characteristics

Mean (SD, range) (*N* = 43 unless otherwise indicated)	
Age	13.56 (1.12, 11.4–15.7)
% male	67%
IQ	84.14 (24.24, 27–129)
ADOS-2 severity	6.05 (2.65, 1–10)
ARI (*n* = 41)	4.51 (3.21, 0–12)
DBC total behaviour problem score (*n* = 41)	53.56 (24.59, 16–127)
SCAS (*n* = 41)	27.90 (17.91, 4–77)
SDQ emotional problems (*n* = 41)	4.29 (2.69, 0–10)
SDQ ADHD symptoms (*n* = 41)	5.15 (2.58, 0–10)
SDQ conduct problems (*n* = 41)	2.12 (1.65, 0–6)

*ADOS-2* Autism Diagnostic Observation Schedule, *ARI* Affective Reactivity Index, *DBC* Developmental Behaviour Checklist, *SCAS* Spence’s Child Anxiety Scale, *SDQ* Strengths and Difficulties Questionnaire

### Parent-rated questionnaires

As ASD is a broad spectrum, we intentionally used a variety of questionnaires to best capture the different types of emotional and behavioural problems exhibited by this population. The details of these are given below.

### Affective Reactivity Index (ARI)

The ARI [51] was used to assess participants’ level of irritability and includes six items relating to feelings/behaviours specific for irritability and one question assessing impairment due to irritability. Internal consistency is reported to be good in samples of young people with ASD (*α* = 0.82) [52].

### Developmental Behaviour Checklist (DBC)

The DBC [53, 54] is a 96-item questionnaire designed to assess emotional and behavioural problems in young people with developmental disabilities and ID. Excellent internal consistency (*α* = 0.94) is reported from large epidemiological samples, along with high correlations (*r* = 0.70–0.86) with other measures of emotional and behavioural disturbance [53, 54].

### Spence’s Child Anxiety Scale (SCAS)

The SCAS [55] is a 38-item questionnaire used to assess current symptoms of anxiety in 6–18-year-olds. Excellent internal consistency (*α* = .92–.93) [56, 57] and convergent validity with DSM-IV-defined anxiety disorders [58] have been reported from samples of young people with ASD.

### Strengths and Difficulties Questionnaire (SDQ)

The SDQ [59] is a 25-item questionnaire used to measure psychiatric symptoms. The SDQ comprises three psychiatric subscales of hyperactivity/inattention (ADHD symptoms), conduct problems and emotional problems (including both anxiety and depression symptoms), along with further subscales of peer-relationship problems and prosocial behaviour. The SDQ maintains good psychometric properties when used with individuals with ID [60] and has been shown to successfully detect change in additional mental health problems following intervention in populations of young people with ASD [61]. Current analyses focused upon the three psychiatric subscales of ADHD symptoms, conduct problems and emotional problems.

### Sensory Experiences Questionnaire 3.0 (SEQ)—brief version for 10–14-year-olds

The SEQ 3.0—brief version for 10–14 year olds (Grace T. Baranek, copyright 2014) is an 18-item questionnaire designed to measure sensory features in young people with ASD. This shortened version, using a subset of items from the original SEQ 3.0, was created specifically for use with the QUEST sample and was based on a factor analysis of the original measure [62]. The SEQ is designed to capture four constructs and enhanced perception, hyper-responsiveness, hypo-responsiveness, and sensory interests, repetitions and seeking behaviour. Comparison of the brief version and the full SEQ found strong correlations between the two for all four constructs (enhanced perception *r* = 0.84, hyper-responsiveness *r* = 0.85, hypo-responsiveness *r* = 0.86, and sensory interests, repetitions and seeking behaviour *r* = 0.81) (Baranek, 2014. unpublished data). However, given the limited number of items measuring each construct in the brief version of the SEQ, the authors recommend grouping responses into two subscales: hyper-responsiveness + enhanced perception and hypo-responsiveness + sensory seeking (Grace T. Baranek, personal correspondence). In previous work with a larger sample (*n* = 311) of 10–14-year-olds with ASD, internal consistency was found to be acceptable for the total score (*α* = 0.75) and for the hyper-responsiveness + enhanced perception subscale (*α* = 0.73), however lower for the hypo-responsiveness + sensory seeking subscale (*α* = 0.64) (Baranek, 2014. unpublished data). In the current total pooled QUEST sample (which included both the extensive sample, and all participants from the intensive sample, including those who completed questionnaire measures but not neurocognitive tasks) (*n* = 198), internal consistency was good for the total score (*α* = 0.85) and the hypo-responsiveness + sensory seeking subscale (*α* = 0.80) and acceptable for the hyper-responsiveness + enhanced perception subscale (*α* = 0.76).

### Direct assessments

#### ASD symptoms

The ADOS-2 [48] is considered a gold-standard instrument for assessing current ASD symptoms and consists of a semi-structured assessment designed to elicit certain ASD behaviours, which are then coded and scored. Based on the total score, a calibrated severity score is calculated, scored 0–10, which takes into account age and language level [63]. A higher score is indicative of a more severe level of ASD symptoms. Participants were assessed with either the ADOS-2 Module 1 (*n* = 2), 2 (*n* = 2), or 3 (*n* = 39), dependent upon their verbal abilities. All ADOS-2 assessments were administered by a trained researcher and co-scored by a second trained researcher, and final scores reflected consensus scores between the two coders.

#### Cognitive ability

IQ was estimated using either the Wechsler Abbreviated Scale of Intelligence (WASI) ([64]) (*n* = 38) or the Wechsler Preschool and Primary Scale of Intelligence (WPPSI) ([65]) (*n* = 5), depending on the child’s age and developmental level. As the WPPSI was used out of age range, age-equivalents were calculated and a ratio IQ derived [ratio IQ = (age equivalent/chronological age) × 100] [66].

### EEG paradigm

#### Stimuli

Auditory stimuli were presented in an oddball paradigm (adapted from [37]). Stimuli were two tones, each of 100 ms in duration with a rise and fall time of 5 ms, and an inter-stimulus interval of 700 ms. The infrequently presented deviant tone (8% probability) consisted of a 1200 Hz tone. The frequently presented standard tone (92% probability) consisted of a 1000 Hz tone. All tones were presented at 70 dB SPL. Stimuli were presented randomly, with the restriction that at least three standard tones (S1, S2 and S3) followed each deviant tone. To avoid substantial differences in trial numbers, analyses focused only on S1, S2 and S3 rather than all standard tones.

### Procedure

Participants were seated within a sound-attenuated EEG suite, where sounds were presented through two speakers, located approximately 1 m in front of the participant. Participants watched two soundless movies whilst the auditory stimuli were presented. High-density scalp EEG was recorded continuously using a 128-channel HydroCel Geodesic Sensor Net system (Electrical Geodesics, Eugene, OR) at a 500-Hz sampling rate, with the NetAmps 400 amplifier which employs a 4 KHz antialiasing filter. Voltages were referenced online to the vertex electrode (Cz). Impedances checked to be below 40 kΩ before recording began. All electrophysiological data were recorded with NetStation 5.1 software (Electrical Geodesics, Eugene, OR), and all tasks were delivered through E-Prime 2.0 experimental design software (Psychology Software Tools, Pittsburgh, PA). Data were stored and analysed offline.

### EEG recording and pre-processing

EEG data were processed offline using BrainVision Analyser 2.0 software (Brain Products, Munich, Germany). Data were down-sampled to 256 Hz, re-referenced to the average reference and filtered using 0.1 Hz high-pass and 30 Hz low-pass infinite impulse response (IIR) phase-shift free Butterworth 24 dB/Oct filters. The data were manually inspected to identify bad channels, which when possible were interpolated using spherical splines. Noisy segments of data were removed by visual inspection prior to running independent component analysis (ICA) ([67]). Visual inspection of the component map was used to identify and remove components representing ocular movement. Semi-automatic artefact detection was subsequently performed to remove any segments with any additional artefacts greater than maximum-minimum values of 200 μV. Epochs of 600 ms, including a − 100 ms prestimulus period, were extracted and averaged for each stimulus category (deviant, S1, S2 and S3). Data were baseline corrected using the 100 ms prior to stimuli presentation.

### ERP analysis

**Fig. 1:**
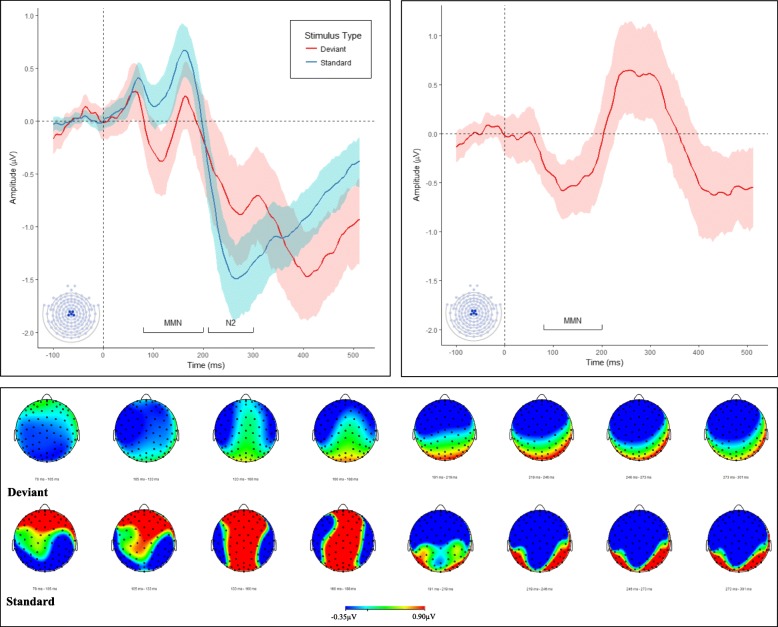
Grand average waveforms to standard and deviant stimuli at Cz montage (*left panel*). Difference wave (response to deviant stimuli—response to standard stimuli) at Cz (*right panel*). Isocontour maps derived from the grand average response to deviant and standard stimuli at 80–300 ms (*bottom panel*). *Shaded areas represent 95% confidence intervals*

The average amount of trials per condition was 68 (SD = 12.85) for all standard stimuli (S1 = 68, S2 = 69 and S3 = 68) and 69 (SD = 12.64) for deviant stimuli. Electrodes of interest were selected based on prior literature [33, 68, 69] and confirmed with visual inspection of the grand average waveform (see Fig. 1). Semi-automatic peak detection was used to mark specific components, and the amplitude and latency of components were extracted for statistical analysis. Each participant’s individual waveform data were inspected to confirm that components of interest fell within the allotted temporal window. The MMN was extracted from a cluster of five electrodes (7, 31, 80, 106, Cz) corresponding to the Cz area. Peak amplitude of the most prominent negative deflection was measured in each participant in the 80–200 ms latency range, consistent with previous literature [19]. Amplitudes for all electrodes in a cluster were averaged.

For MMN analysis, responses to S1, S2 and S3 were averaged, and analyses compared response to deviant vs. standard stimuli. For analysis of habituation, responses to the first (S1), second (S2) and third (S3) standard tone after a deviant tone were averaged separately. From inspection of the grand averages (Fig. 1), it was clear that the ERP response to stimuli was characterised by two negative deflections, one early and one late. Thus, habituation analyses were conducted not only at the early N1 component (using the same latency window as was used in the MMN analysis, 80–200 ms), but also a later negative component (N2, 210–300 ms). Peak amplitude of the most prominent negative deflection in these latency ranges for S1, S2 and S3 was measured in each participant.

### Analytic strategy

All analyses were completed in Stata 14 [70]. To ensure that the paradigm had reliably elicited the MMN component, amplitudes to deviant and standard tones at the early component were compared using planned pairwise comparisons. MMN amplitude was measured as the difference waveform obtained by subtracting response to the standard tones from response to the deviant tones. A habituation index was measured as the difference waveform obtained by subtracting response to S1 from response to S3. A higher value indicates a greater decrease in ERP response between S1 and S3 (i.e., greater habituation). Where significant associations were found with the habituation index, planned follow-up analyses looked at responses to each standard tone (S1, S2, S3) separately to clarify whether response to a specific standard tone was driving effects. Before beginning analyses, data were checked for skewness and outliers. As the S1 and S3 variables were negatively skewed, they were square root transformed. Outliers in EEG data were identified using box and whisker plots (Stata command graph box). This identifies outliers as values outside 1.5 × the interquartile range ± the value of the upper/lower quartile [71]. One outlier was identified in the MMN difference wave data and two outliers in the habituation index data. These were removed before conducting each analysis. For completeness, analyses were also conducted on the full dataset (including outliers). These are reported in Additional file 1, along with additional post hoc analyses adjusting for the overall number of available trials per participant, and using the mean, rather than peak, ERP amplitude.

Bi-variate correlations were calculated between all parent-rated predictor variables and EEG outcome variables to gain an initial understanding of the data. These are listed in Additional file 1. Following this, analyses used multivariate regression to test for an association between ERP response and SDQ subscales of emotional problems and ADHD symptoms and conduct problems, along with the ARI irritability scale. A separate regression was run to test for an association between ERP response and DBC total behaviour problem score. Questionnaires were grouped in this manner in the analyses as the SDQ and ARI were developed in non-ASD populations and demarcate well-defined domains of psychopathology (e.g., emotional problems, ADHD, conduct problems, irritability). Conversely, the DBC was designed for people with developmental disorders, including ASD and ID. Here, the total score indexes a range of emotional and behavioural problems which are often found in individuals with developmental disorders. The multivariate approach was selected as it is statistically parsimonious and takes account of multiple testing amongst correlated outcomes. Where trend or significant associations were found, results were first adjusted for age, sex and IQ, and then for age, sex, IQ and ASD severity, as measured by the ADOS calibrated severity score. Two separate sensitivity analyses were conducted, first excluding those using medication known to affect brain functioning (*n* = 5) and second excluding those with epilepsy (*n* = 2). Finally, to assess how brain indices related to real-life sensory sensitivities, bi-variate correlations were computed between key ERP components and the two SEQ subscales.

## Results

### Perceptual sensitivity as measured by the MMN

The ERP response to deviant tones was significantly greater than the response to the standard tones (mean standard amplitude = − 0.39, SD = 0.78, range − 3.40–1.27; mean deviant amplitude = − 0.93, SD = − 1.07, range − 3.71–1.11; *t*(42) = 3.90, *p* < 0.01), confirming the presence of the MMN.

**Fig. 2:**
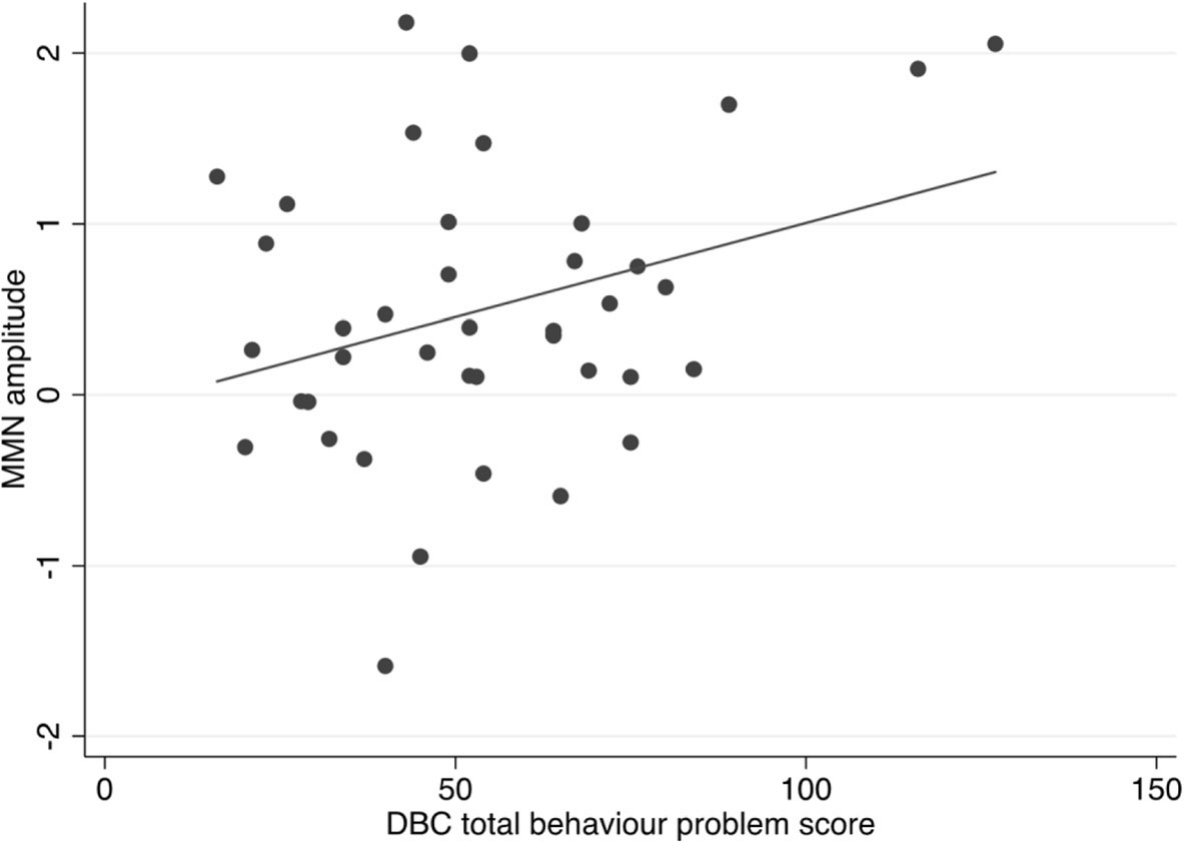
Association between behaviour problems, rated by the Developmental Behavior Checklist, and MMN difference wave (response to deviant stimuli—response to standard stimuli)

No significant associations were found between the SDQ subscales or ARI total and MMN amplitude (*ps* = 0.22–0.99). A significant association was found between MMN amplitude and DBC total behaviour problem score (*β* = 9.51, *p* < 0.05), and this association remained at a trend level when controlling for age, sex and IQ (*β* = 9.40, *p* = 0.07), but became non-significant when controlling for age, sex, IQ and ASD severity (*β* = 8.77, *p* = 0.11). The association remained significant in sensitivity analyses, first excluding those using medication (*β* = 10.10, *p* < 0.05), and then excluding participants with epilepsy (*β* = 10.39, *p* < 0.05). Figure 2 depicts the association between MMN amplitude and DBC total behaviour problem scores, in that those with greater MMN amplitude had higher DBC total behaviour problem scores. This association was not specifically driven by response to either standard or deviant ones as neither was significantly associated with DBC total behaviour problem score (*p* = 0.18 and *p* = 0.78 respectively).

### Habituation

No significant association was found between behaviour and the habituation index at the early N1 component (*ps* = 0.09–0.99).

At the later N2 component, the SDQ emotional problem subscale was positively associated with the habituation index (*β* = 1.47, *p* < 0.01), in that those with higher habituation had a greater SDQ emotional problems score, and this association remained when controlling for age, sex and IQ (*β* = 1.77, *p* < 0.01) and controlling for age, sex, IQ and ASD severity (*β* = 1.80, *p* < 0.01), and in sensitivity analyses excluding participants using medication (*β* = 1.53, *p* < 0.01), and excluding participants with epilepsy (*β* = 1.49, *p* < 0.01). No association was found between the habituation index and the other SDQ subscales, ARI total, DBC total behaviour problem score (*ps* = 0.16–0.55).

Given that the directionality of association between habituation and anxiety was not what was expected (hypotheses predicted *decreased* habituation would be associated with greater anxiety, but in instead, the opposite was found), validation analyses were conducted with other measures of anxiety that were available. A comparable significant association was found with the SCAS total (*β* = 9.01, *p* < 0.01), and this remained when adjusting for age, sex and IQ (*β* = 10.64, *p* < 0.01), and age, sex, IQ and ASD severity (*β* = 10.71, *p* < 0.01), and when excluding participants using medication (*β* = 9.36, *p* < 0.01), and excluding participants with epilepsy (*β* = 9.12, *p* < 0.01).

### Response to S1, S2 and S3

**Fig. 3:**
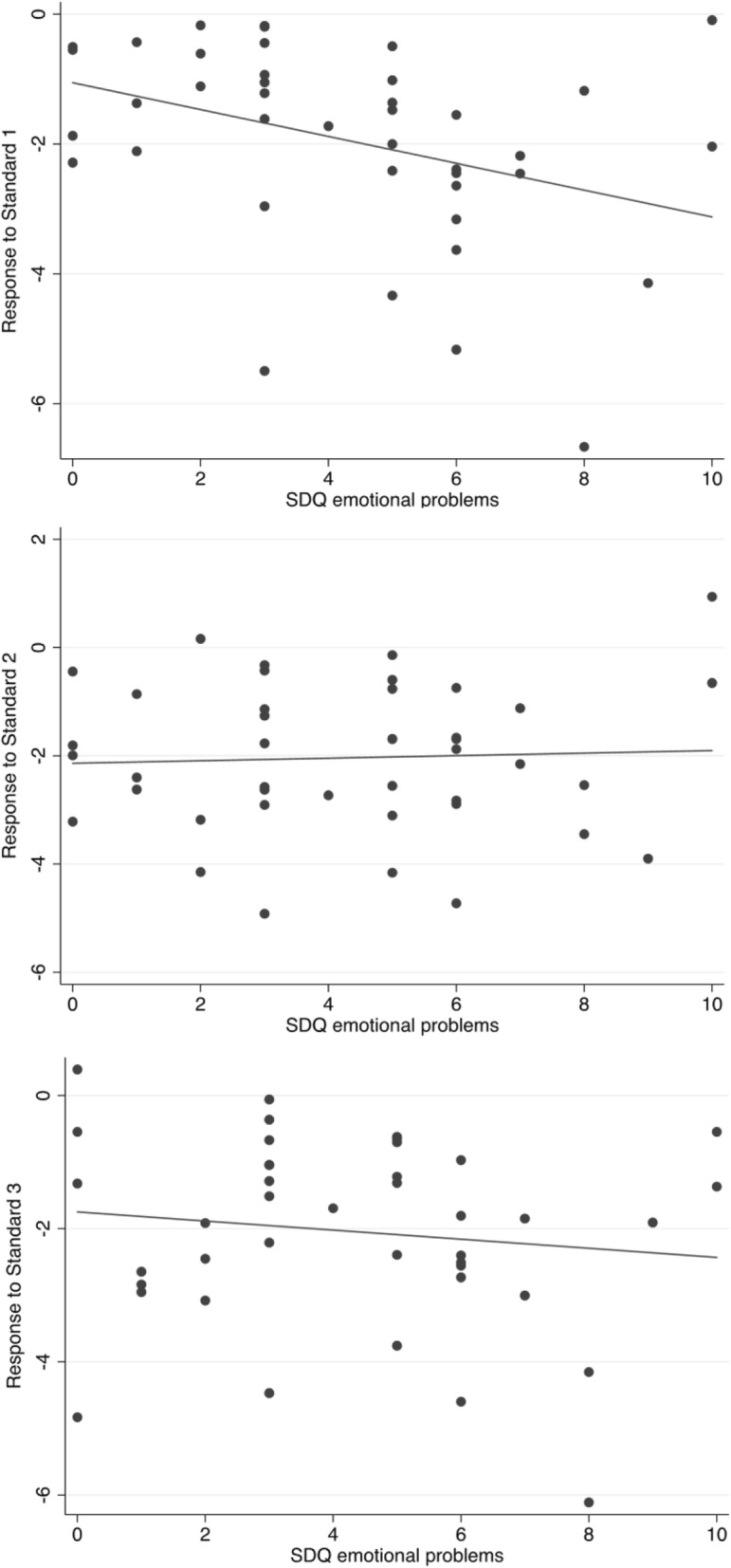
Association between emotional problems, as rated by the Strengths and Difficulties Questionnaire, and N2 amplitude to the first (S1), second (S2) and third (S3) standard presented directly after a deviant stimulus

To aid in the interpretation of the association between emotional problems and habituation, analyses next tested how SDQ emotional problems predicted response to S1, S2 and S3. There was a selective association with S1, in that higher levels of SDQ emotional problems were associated with greater S1 amplitude (*β* = 2.09, *p* < 0.05), but were not associated with the S2 (*p* = 0.78) or S3 (*p* = 0.32) (see Fig. 3). This association with S1 remained significant when controlling for age, sex and IQ (*β* = 2.60, *p* < 0.05), controlling for age, sex, IQ and ASD severity (*β* = 2.65, *p* < 0.05), and when excluding those using medication (*β* = 2.06, *p* < 0.05), and participants with epilepsy (*β* = 1.87, *p* < 0.05). The same selective association with S1 was found using the SCAS (*β* = 17.53, *p* < 0.01) and remained in all covariation and sensitivity analyses. Post estimation tests (controlling for age, sex, IQ and ASD severity) found the SDQ emotional problems—S1 association was not significantly different as compared against the SDQ emotional problems—S2 association (*p* = 0.65), but was at a trend level when compared against the SDQ emotional problems—S3 association (*p* = 0.07). The SCAS—S1 association was at a trend level when compared against the SCAS—S2 association (*p* = 0.07), and significantly different when compared against the SCAS—S3 association (*p* = 0.03).

Thus, although analyses began with a focus on habituation, results suggest that the habituation-anxiety association was likely driven by a selective association between anxiety symptoms and the first standard stimulus presented after the deviant stimulus.

### Correlations between key ERP components and parent-rated sensory sensitivities

A trend positive correlation was found between MMN amplitude and the SEQ hyper-responsiveness + enhanced perception subscale (*r* = 0.29, *p* = 0.07). The correlation between MMN amplitude and the SEQ hypo-responsiveness + sensory seeking subscale was non-significant (*r* = 0.25, *p* = 0.12). No significant correlations were found between N2 response to S1 and either SEQ subscale.

## Discussion

This study investigated whether alterations in neural indices of perceptual processing and discimination were associated with emotional and behavioural problems in young people with ASD. Results showed that increased sensitivity to deviant stimuli was associated with increased behaviour problems, whereas heightened response to standard stimuli following a deviant stimulus was associated with increased emotional problems, and this appeared to be mainly driven by anxiety symptoms.

The current finding of increased sensitivity to deviant stimuli, as measured by MMN amplitude, being associated with higher levels of challenging behaviours, as rated by the DBC total behaviour problem score, builds on prior work that has found comparable relationships in ASD populations using care-giver ratings of perceptual sensitivity [38–40]. In the current study, the association remained at a trend when adjusting for age, sex and IQ, and in sensitivity analyses excluding those taking psychotropic medication and those with a diagnosis of epilepsy. However, the association became non-significant when ASD severity was also accounted for, in addition to age, sex and IQ. Nevertheless, when ASD severity was added as a covariate, the standardised coefficients were not drastically changed, dropping from 9.51 to 8.77, and this change may be due to an increase in standard error with the inclusion of an additional covariate. Theoretically, the overlap between MMN amplitude and ASD severity is unsurprising given that sensory atypicalities are part of the diagnostic criteria for ASD, and MMN amplitude appeared to be tapping some form of sensory sensitivity, as shown by the trend correlation with the SEQ hyper-responsiveness + enhanced perception subscale. It is not possible to know from cross-sectional data, as was used as the current study, whether higher ASD severity leads to more atypical perceptual processing, or vice versa. It should also be noted that the few individuals with particularly high levels of reported behaviour problems (an established clinical characteristic of individuals with ASD; [7]) could have substantially contributed to the reported association between MMN amplitude and DBC behaviour problems (see Fig. 2). The present study is unable to disentangle whether the association between MMN amplitude and behaviour problems only applies to individuals with particularly high levels of behaviour problems (in a categorical manner) or is relevant to individuals with ASD with a range of behaviour problems (in a continuous manner). This requires further examination in a larger sample.

Additionally, results showed that the association with MMN amplitude was not driven by response to either the standard or the deviant in isolation, but the relative difference in neural response between the two (i.e., discrimination). Given that the MMN is correlated with individual discrimination ability [19–21], and is a relatively early component in the processing pathway, these results suggest that early, pre-attentive sensitivity to changes in perceptual input may be an important factor to consider in the aetiology of co-occurring psychopathology in individuals with ASD. Additionally, the MMN response appeared to be tapping perceptual processes that related to real-life sensory sensitivities, as shown by the association with the parent-rated SEQ subscale that indexed hyper-responsiveness and enhanced perception. Results are thus in line with clinical guidelines that recommend taking into account individual sensory sensitivities when designing interventions for use with young people with ASD [72]. However, it should be held in mind that the DBC is a broad-brushstroke measure and indexes a variety of types of challenging behaviours. From the association with the DBC total behaviour problem score, it cannot be determined exactly what type of behavioural problems hyper-sensitivity to perceptual input relates to, as prior literature has found associations to a variety of difficulties [39–41, 43, 44].

Although analyses began with showing that increased habituation was associated with increased emotional problems, this appeared to be driven by a selectively greater neural response to the first standard stimulus (S1) following a deviant stimulus. It should be stressed that these analyses were exploratory and require replication, as the results were not hypothesised a priori. However, a comparable association was found using multiple measures of anxiety, suggesting first that this was unlikely to be due to a type 1 error and second that the association with the SDQ emotional problems subscale was likely to be driven by items indexing anxiety. Current findings build on prior work, which has used questionnaire ratings to find associations between sensory over-responsivity and anxiety in individuals with ASD [15, 41–44]. Thus, although speculative, results are interpretable using the ‘intolerance of uncertainty’ framework [73], which has been conceptualised as a tendency to react negatively to uncertain situations and events [74]. Higher levels of parent and self-rated intolerance of uncertainty have been found in children and adolescents with ASD as compared to typically developing youth [73, 75, 76], and in both ASD and typically developing youth, greater intolerance of uncertainty are associated with higher levels of parent-rated anxiety, as measured by the SCAS [73]. In addition to the link between intolerance to uncertainty and anxiety, research has found that sensory sensitivity is related to both of these concepts [75, 77, 78]. Thus, in the current study when uncertainty was introduced (by the deviant stimuli), this may have led to a heightened state of arousal in participants who were rated as being more anxious. Conversely, biases in local perceptual processing may have led the incoming stimuli being perceived as more unpredictable, thus provoking heightened anxiety in participants with these perceptual biases. These interpretations are supported by existing literature, for example where temporally unpredictable auditory stimuli have been found to induce anxiety in mice and humans [47], and biases in perceptual processing (specifically in hyper-sensitivity to local details) are associated with greater levels of compulsive-like behaviours (for example insistence on sameness) in children [79]. We propose the hyper-arousal induced by uncertainty was captured by the increased neural response to stimuli presented directly after the deviant (S1), but once it was recognised as one of the standard repeated stimuli, arousal decreased, thus explaining the lack of effect for S2 or S3. However, currently, the field remains unclear about directionality of pathways between sensory processing/sensitivities, intolerance of uncertainty and anxiety [75, 77]. The present data cannot make claims about the directionality of effects or indeed if a different, unacknowledged factor is driving the association between these concepts. It should be noted that the current study did not have a measure intolerance of uncertainty, and so, the link to this concept is speculative at present. Future work should use comprehensive measures of intolerance of uncertainty and follow a priori hypotheses, to better disentangle pathways between sensory sensitivity, intolerance of uncertainty and anxiety, in individuals with ASD.

### Strengths and limitations

The first strength is the novelty of the approach taken. There is a limited body of research focused on understanding how variation in neurocognitive functioning may underpin variation in the behavioural phenotype of ASD, and despite the high prevalence of emotional and behavioural problems in individuals with ASD [1, 2, 4, 5], there is a paucity of ASD-specific models of psychopathology. Given the persistence of this psychopathology in youth with ASD [80], research is required to understand how to best predict and treat these co-occurring problems. Although previous work has looked at characteristics such as IQ, age, sex and ASD severity, few studies have looked specifically at domains of cognitive functioning. Focusing on carefully selected cognitive domains such as perceptual processing gives a deeper understanding of potential drivers of psychopathology beyond that of broad characteristics such as IQ and age and may offer clues as to the specific neurocognitive mechanisms at play. Additionally, the current study builds on prior work that has used parent-report of both cognitive functioning and behaviour, where shared method variance may have contributed to significant associations.

Another strength is the use of a community sample, where the target population was all individuals with a diagnosis with ASD in a specified geographical area (as opposed to using an opportunity sample of individuals with ASD who present to clinic with emotional and behavioural difficulties), thus making the sample more representative of ASD as a whole. Although the sample who completed EEG assessments had a higher IQ (as was expected) and lower scores on the SEQ hypo-responsiveness + sensory seeking subscale than the full sample, in all other key descriptive variables (ASD severity, age, co-occurring mental health problems), there were no significant differences (see Additional file 1). The sample also deliberately over-sampled females, meaning we had increased power to detect sex differences (unlike many other studies). Finally, the use of EEG meant that completing the paradigm did not require an overt response and thus allowed collection of data from a broader sample of participants (IQ range of 27–129). This approach is in line with recent commentaries calling for the inclusion of historically understudied populations within ASD [81].

In terms of limitations, the current study only measured one type of perceptual processing, and future research is needed to investigate if hyper-sensitivities in other modalities (e.g., proprioceptive, vestibular) are also associated with emotional and behaviour problems in individuals with ASD. Additionally, although the primary research aim was to test which neurocognitive domains are associated with psychopathology within individuals with ASD, the lack of control groups limits interpretation of results. Whether similar associations between cognition and behaviour are found in non-ASD samples, or if the current associations are specific to ASD, remains a question for future research. A further limitation of the current work is the use of a moderately sized sample, which could have led to limited power to detect associations of smaller effect. Current analyses also included multiple statistical tests, which should be held in mind when interpreting results, especially those that were not hypothesised a priori. Further work is required using larger sample sizes, to allow for more rigorous statistical testing and potential replication of the unpredicted results.

### Implications

Current results suggest that alterations in sensory processing and discrimination could be considered as potential drivers of co-occurring emotional and behaviour problems in individuals with ASD (although this requires empirical testing using longitudinal studies, including studies starting at a younger age). Clinically, a comprehensive sensory assessment could be helpful when planning interventions with individuals with ASD and challenging behaviours and anxiety symptoms. Although surveys have found sensory-based interventions are commonly used in individuals with ASD [82], the specific targets of sensory interventions often differ, along with the methodologies used. Better characterisation of perceptual processing atypicalities in individuals with ASD would guide the development of more targeted interventions. The present results also suggest that a focus on intolerance of uncertainty may be helpful, especially as there is some preliminary evidence to suggest interventions targeting this concept may be efficacious in typically developing adolescents with anxiety disorders [83, 84].

## Conclusions

The current study highlights how specific aspects of perceptual processing and discrimination are associated with the presence of additional emotional and behavioural problems in young people with ASD. Although the directionality of the pathway between cognition and behaviour cannot be assessed without longitudinal designs, the current work suggests alterations in perceptual processing and discrimination are important to consider when formulating a mechanistic understanding of additional psychopathology in people with ASD. This in turn will inform the design of novel, targeted interventions, and improve long-term outcomes for people with ASD.

## Additional file

Additional file 1: Supplementary materials (DOCX 87 kb)

## Abbreviations

ADI: Autism Diagnostic Interview
ADOS: Autism Diagnostic Observation Schedule
ARI: Affective Reactivity Index
ASD: Autism spectrum disorder
DBC: Developmental Behavior Checklist
EEG: Electroencephalography
ERP: Event-related potential
ID: Intellectual disability
IQ: Intelligence quotient
MMN: Mismatch negativity
S1: First standard after the deviant tone
S2: Second standard after the deviant tone
S3: Third standard after the deviant tone
SCAS: Spence’s Child Anxiety Scale
SDQ: Strengths and Difficulties Questionnaire
SEQ: Sensory Experiences Questionnaire (brief version)
WASI: Wechsler Abbreviated Scale of Intelligence
WPPSI: Wechsler Preschool and Primary Scale of Intelligence
