# Supplementary material for: Alterations in electrophysiological indices of perceptual processing and discrimination are associated with co-occurring emotional and behavioural problems in adolescents with autism spectrum disorder
Source: Mol Autism. 2018 Oct 5;9:50. doi: 10.1186/s13229-018-0236-2 (PMC6173917; doi:10.1186/s13229-018-0236-2)
Supplement: Supplementary file 1 — Supplementary materials (DOCX 87 kb) [file 13229_2018_236_MOESM1_ESM.docx]

**Supplementary Materials 1 – Details of QUEST sample**

Participants were part of a longitudinal community sample recruited at age 4-8 years [1]. The target population for the study was all children born between 01/09/2000 and 31/08/2004, living in two London boroughs (one inner and one outer London), who had received a clinical diagnosis of ASD. Secondary care services and local autism support groups identified 447 children as being eligible for the study. Clinical diagnoses of ASD were established by local multidisciplinary teams, led by a community paediatrician, using structured assessments such as the Autism Diagnostic Interview-Revised [ADI-R; 2], the Developmental, Dimensional and Diagnostic Interview [3DI; 3], the Diagnostic Interview for Social and Communication Disorders [DISCO; 4] and the Autism Diagnostic Observation Schedule – Generic [ADOS-G; 5]. A total of 277 children were successfully recruited into the study at age 4-8 years. ASD symptomatology was assessed with the Social Communication Questionnaire - Lifetime Version [SCQ; 6]; all cases with a low SCQ score (total<10; n=28) were reviewed with clinicians and their ASD diagnoses confirmed. At this point, the sample was split into an Extensive subsample (n=176) that received the core assessments, and an Intensive subsample (n=101) that received a more detailed assessment. All participating girls were invited into the Intensive subsample in order to make sex comparisons possible, as well as a random selection of boys, stratified to provide equal numbers on IQ (</> 70), borough (inner/outer London), age (</> 6.8 years) and SCQ score (</>22). The sample was followed-up at age 11-15 years, and the Extensive/Intensive sampling design employed at age 4-8 was retained. 76% (n=211) of the original sample (n=277) participated in the follow-up: 128 participants formed the Extensive Wave 2 subsample and 83 participants formed the Wave 2 Intensive subsample. Within the Intensive subsample ASD diagnosis was confirmed using the ADOS-2 [7]. Based on information from previous data sets of young people with ASD, rules were set by the research project’s lead statistician regarding whether the ADI-R should also be administered. The ADI-R was required in 66/83 cases. All participants were above the diagnostic cut-offs for autism or ASD on either or both the ADOS-2 and the ADI-R using recommended cut-offs [2, 8]. Of the 83 participants within the Intensive subsample, 53 completed some combination of the neurocognitive tasks (including the n=43 who completed the auditory oddball paradigm outlined in the main manuscript). See Supplementary Figure 1 for a breakdown of the sampling strategy.

**Participated in Wave 1**

N = 277 (62.0% of target population)

82.0% male

Selected for Wave 1 Intensive assessment

n=131

Did not participate in Intensive assessment

Assessment completed

**Intensive Sample**

n=101

56% male (57 male, 44 female)

**Extensive Sample**

N=176

97% male (170 male, 6 female)

**Eligible for Wave 2**

N=277 (all Wave 1 ppts)

**Extensive sample participation**

N= 128 (73% of Wave 1 ppts)

96% male (123 male, 5 female)

**Intensive sample participation**

N= 83 (82% of Wave 1 ppts)

57% male (47 male, 36 female)

**Completed some form of neurocognitive assessment**

N= 53 (64% of intensive sample)

62% male (33 male, 20 female)

**Target population**

Children with an ASD diagnosis, born 01/09/2000- 01/09/2004, living in Bromley or Lewisham

N=447

**Figure S1. Summary of QUEST Sample Recruitment and Selection**

**Supplementary Materials 2 - Sample Characteristics and Attrition Analysis of Intensive Sample at Wave 2**

| **Item Mean (SD; range)** | **A:**  **Whole Sample (Intensive + Extensive)** | **B:**  **Intensive Sample** | **C:**  **Intensive Sample Who Only Completed Auditory Oddball Paradigm** | **Test of group means**  **A vs. C** | **Test of group means**  **B vs. C** |
| --- | --- | --- | --- | --- | --- |
| **Time 1 Data (4-8 years)** | **(n=277)** | **(n=101)** | **(n=43)** |  |  |
| **IQ** | 72.58 (26.53; 19-129) | 66.06 (27.92; 19-120) | 79.80 (20.84; 25-120) | A<C* | B<C** |
| **SCQ** | 20.09 (7.51; 1-42) | 19.98 (7.10; 3-34) | 19.23 (19.23; 3-33) | ns | ns |
| **DBC total behaviour problem score** | 71.16 (29.61; 6-141) | 72.69 (28.17; 13-129) | 78.26 (30.76; 13-139) | ns | ns |
| **Time 2 Data (10-15 years)** | **(n=211)** | **(n=83)** | **(n=43)** |  |  |
| **IQ** | - | 67.40 (32.40; 19-129) | 84.14 (23.24; 27-129) | - | B<C** |
| **Age** | 13.51 (1.12; 11-2.15.8) | 13.45 (1.13; 11.4-15.7) | 13.56 (1.12; 11.4-15.7) | ns | ns |
| **SCQ** | 17.70 (6.91; 3-25) | 17.24 (6.51;  4-31) | 16.26 (5.73; 5-27) | ns | ns |
| **ADOS-2** | - | 6.57 (2.61;  1-10) | 6.05 (2.65; 1-10) | - | ns |
| **% Male** | 82% | 57% | 67% | A<C* | ns |
| **SDQ emotional problems** | 4.16 (2.65; 0-10) | 3.92 (2.48;  0-10) | 4.29 (2.69; 0-10) | ns | ns |
| **SDQ ADHD** | 5.94 (2.62; 0-10) | 5.36 (2.42;  0-10) | 5.15 (2.58; 0-10) | ns | ns |
| **SDQ conduct problems** | 2.32 (1.84; 0-8) | 2.12 (1.67;  0-8) | 2.12 (1.65; 0-6) | ns | ns |
| **ARI** | 4.56 (3.59; 0-12) | 4.53 (3.40;  0-12) | 4.51 (3.21; 0-12) | ns | ns |
| **DBC total behaviour problem score** | - | 56.52 (25.83; 3-127) | 53.56 (24.59; 16-127) | - | ns |
| **SCAS** | - | 24.79 (17.07; 1-77) | 27.90 (17.91; 4-77) | - | ns |
| **SEQ hyper-responsiveness + enhanced perception** | 2.88 (0.87; 1-4.78) | 2.88 (0.81;1-4.78) | 2.86 (0.81; 1-4.78) | ns | ns |
| **SEQ hypo-responsiveness + sensory seeking** | 2.09 (0.80; 1-4.78) | 2.05 (0.75; 1-3.67) | 1.82 (0.62; 1-3.13) | A>C** | B>C* |

ADOS-2 Autism Diagnostic Observation Schedule; ARI Affective Reactivity Index; DBC Developmental Behaviour Checklist; SCAS Spence’s Child Anxiety Scale; SCQ Social Communication Questionnaire; SDQ Strengths and Difficulties Questionnaire; SEQ Sensory Experiences Questionnaire – Brief Version. – indicates data was not collected from extensive sample at Time 2. ***p*<0.01, **p*<0.05

|  | **1** | **2** | **3** | **4** | **5** | **6** | **7** | **8** |
| --- | --- | --- | --- | --- | --- | --- | --- | --- |
| **SDQ Emotional Problems (1)** | **-** |  |  |  |  |  |  |  |
| **SDQ Conduct Problems (2)** | .29* | - |  |  |  |  |  |  |
| **SDQ ADHD Symptoms (3)** | .23 | .27* | - |  |  |  |  |  |
| **ARI total (4)** | .43** | .53** | .22 | - |  |  |  |  |
| **DBC total behaviour problem score (5)** | .38** | .56** | .62** | .63** | - |  |  |  |
| **SCAS total (6)** | .77** | .19 | .17 | .35** | .31** | - |  |  |
| **SEQ Hyper + Enhanced Perception (7)** | .40** | .31* | .40** | .49** | .63** | .21 | - |  |
| **SEQ Hypo + Sensory Seeking (8)** | .26 | .35* | .36** | .34* | .67** | .19 | .64** | **-** |
| **MMN Difference Wave (9)** | .01 | .20 | .06 | .17 | .32* | -.03 | .29 | .25 |
| **Habituation Index (10)** | .60** | .10 | .23 | .23 | .22 | .53** | -.01 | -.01 |
| **S1 N2 Amplitude (11)** | .36* | .08 | -.03 | .06 | -.07 | .46** | -.17 | -.06 |
| **S2 N2 Amplitude (12)** | .05 | .06 | .25 | .09 | .27 | -.17 | .34* | .15 |
| **S3 N2 Amplitude (13)** | .16 | .08 | -.19 | .10 | -.09 | .18 | -.01 | .06 |

**Supplementary Materials 3 – Bi-Variate Correlations of All Key Parent-Rated Questionnaire and EEG Variables**

ARI Affective Reactivity Index; DBC Developmental Behaviour Checklist; SCAS Spence’s Child Anxiety Scale; SDQ Strengths and Difficulties Questionnaire; SEQ Sensory Experiences Questionnaire – Brief Version. ***p*<0.01, **p*<0.05

**Supplementary Materials 4 – Multivariate Analyses Conducted On Full Sample Including Outliers**

**MMN Analysis**

When outliers were included, the association between MMN amplitude and DBC total behaviour problem score became non-significant (β=6.19, *p*=0.15; see Supplementary Figure 2), and remained so when controlling for age, sex and IQ (β=7.09, *p*=0.15), and for age, sex, IQ and ASD severity (β=6.48, *p*=0.22). The association remained non-significant in sensitivity analyses, first excluding those using medication (β=0.52, *p*=0.15), and then excluding participants with epilepsy (β=6.92, *p*=0.12). No association was found between MMN amplitude and the SDQ subscales or ARI total.

**Supplementary Figure 2. Association between Behaviour Problems, rated by the Developmental Behavior Checklist, and MMN Difference Wave.** Statistical outliers in MMN difference wave variable are highlighted.

**Habituation Analyses**

When outliers were included, the SDQ emotional problems subscale and habituation index association remained at a trend level of significance (β=0.69, *p*=0.05; see Supplementary Figure 3), and became significant when controlling for age, sex and IQ (β=0.89, *p*<0.05) and age, sex, IQ and ASD severity (β=0.96, *p*<0.05). The association dropped to a trend level of significance in sensitivity analyses excluding participants using medication (β=0.68, *p*=0.07), and but remained significant excluding participants with epilepsy (β=0.70, *p*<0.05). No association was found between the habituation index and the other SDQ subscales, ARI total, DBC total behaviour problem score.

**Supplementary Figure 3. Association between SDQ Emotional Problems and Habituation Index.** Statistical outliers in habituation index variable are highlighted.

**Supplementary Materials 5 – Multivariate Analyses Controlling for Number of Available Trials Per Participant**

When analyses controlled for the number of trials overall each participant had available, the association between MMN amplitude and DBC total behaviour problem score became a non-significant trend (β=7.98, *p*=0.09), the SDQ emotional problems subscale and habituation index association remained significant (β=1.43, *p*<0.01), and the SDQ emotional problems association with S1 amplitude remained at a trend level of significance (β=1.76, *p*=0.05).

**Supplementary Materials 6 – Multivariate Analyses Using Mean ERP Amplitude**

We found strong correlations between mean and peak ERP amplitudes (*r*=0.89 for the MMN, *r*=0.95 for the habituation index, *r*=0.98 for S1, *r*=0.95 for S2 and *r*=0.96 for S3). We find the same pattern of results as are reported in the main manuscript, where the peak ERP amplitude was used. See below for details.

**MMN Analyses**

A significant association was found between MMN mean amplitude and DBC total behaviour problem score (β=9.97, *p*<0.05), and this association remained significant when controlling for age, sex and IQ (β=9.77, *p*<0.05), but dropped to a trend when controlling for age, sex, IQ and ASD severity (β=9.24, *p*=0.07). The association remained significant in sensitivity analyses, first excluding those using medication (β=10.57, *p*<0.05), and then excluding participants with epilepsy (β=11.01, *p*<0.05).

**Habituation/S1 Analysis**

At the later N2 component, the SDQ emotional problems subscale was positively associated with the mean habituation index (β=1.29, *p*<0.01), in that those with higher habituation had a greater SDQ emotional problems score, and this association remained when controlling for age, sex and IQ (β=1.59, *p*<0.01) and controlling for age, sex, IQ and ASD severity (β=1.67, *p*<0.01), and in sensitivity analyses excluding participants using medication (β=1.34, *p*<0.01), and excluding participants with epilepsy (β=1.27, *p*<0.01). There was a selective association with S1 mean amplitude, in that higher levels of SDQ emotional problems were associated with greater S1 amplitude (β= -.92, *p*<0.05), and this remained in all covariation and sensitivity analyses (all *ps*<0.05).

**References**

1. Salazar F, Baird G, Chandler S, Tseng E, O'sullivan T, Howlin P, Pickles A, Simonoff E: Co-occurring emotional and behavioral disorders in preschool and elementary school-aged children with autism spectrum disorder. J Autism Dev Disord 2015, 45:2283-2294.

2. Rutter M, Le Couteur A, Lord C: Autism Diagnostic Interview-Revised. Los Angeles, CA: Western Psychological Services; 2003.

3. Skuse D, Warrington R, Bishop D, Chowdhury U, Lau J, Mandy W, Place M: The developmental, dimensional and diagnostic interview (3di): a novel computerized assessment for autism spectrum disorders. J Am Acad Child Adolesc Psychiatry 2004, 43:548-558.

4. Wing L, Leekam SR, Libby SJ, Gould J, Larcombe M: The diagnostic interview for social and communication disorders: Background, inter‐rater reliability and clinical use. J Child Psychol Psychiatry 2002, 43:307-325.

5. Lord C, Risi S, Lambrecht L, Cook Jr EH, Leventhal BL, DiLavore PC, Pickles A, Rutter M: The Autism Diagnostic Observation Schedule—Generic: a standard measure of social and communication deficits associated with the spectrum of autism. J Autism Dev Disord 2000, 30:205-223.

6. Rutter M, Bailey A, Lord C: Social Communication Questionnaire. Los Angeles, CA: Western Psychological Services; 2003.

7. Lord C, Rutter M, DiLavore PC, Risi S, Gotham K, Bishop SL, Luyster RJ, Guthrie W: The Autism Diagnostic Observation Schedule, Second Edition (ADOS-2). San Antonio, TX: Pearson Assessments; 2012.

8. Risi S, Lord C, Gotham K, Corsello C, Chrysler C, Szatmari P, Cook EH, Leventhal BL, Pickles A: Combining information from multiple sources in the diagnosis of autism spectrum disorders. J Am Acad Child Adolesc Psychiatry 2006, 45:1094-1103.
